# Leveraging Promising Policies to Support Long-Term Care Residents' Quality of Life Post-Pandemic

**DOI:** 10.1093/geroni/igab046.1425

**Published:** 2021-12-17

**Authors:** Deanne Taylor, Janice Keefe, Heather Cook

## Abstract

Long-term care (LTC) is highly regulated and often the policy language is complex and in tension with residents’ quality of life goals. Prior to COVID-19, LTC policy levers prioritized safety over other quality domains such as privacy, dignity, spirituality, and comfort. During the pandemic, this focus on safety regulations, while important, intensified in ways that often negatively impacted residents’ overall quality of life. In this symposium, we share findings from a five- year research project where we conducted a unique and expansive review of regulatory policy across four Canadian jurisdictions. We highlight how 11 different quality of life domains are supported and which texts offering promising policy language to enhance a well-rounded quality of life for residents. These are timely insights to offer as policy-makers look to the future and consider the lessons learned from the pandemic. We contend that creating more LTC policy is not a timely pathway forward to LTC reform. Instead, we suggest that existing policy can be leveraged when applied within a resident-centred quality of life lens. We will guide attendees through examples of existing promising policies highlighting how they might leveraged in planning for a better LTC system. The discussion will be rooted in our unique resident-centred approach to policy analysis using specific domains of quality of life and then applied to four different perspectives: residents, families, staff and volunteers. Our discussant a Ministry of Health decision-maker will address the implications of our research for post-pandemic planning to improve resident quality of life

